# Taming COVID-19 by Regulation: An Opportunity for Self-Reflection

**DOI:** 10.1017/err.2020.43

**Published:** 2020-04-24

**Authors:** Alberto ALEMANNO

**Affiliations:** * Jean Monnet Professor of European Union Law, HEC Paris, Editor-in-Chief of the *European Journal of Risk Regulation* (EJRR) and Editor of the EJRR Special Issue “Taming COVID-19 by Regulation”; email: alemanno@hec.fr.

The COVID-19 outbreak is not the first nor last series of recent real or potential catastrophes – be they natural disasters, terrorist attacks or pandemics – that have taken by surprise governments, globalised firms and the citizenry.^1^

Yet, due to its near-unprecedented impact on the highly interconnected but vulnerable systems that define the modern world, this pandemic has been testing our ability to govern risk more than any other crisis before. The last time the world responded to a global emerging disease epidemic of the scale of the current novel coronavirus without having access to vaccines was the 1918–1919 H1N1 influenza pandemic.^2^ Ironically, the measures mobilised today to counter COVID-19 – the so-called “non-pharmaceutical interventions”^3^ – are essentially the same as those deployed a century ago, and that despite significant social, technological as well as governance differences between 1918 and today.^4^

No other emergency led to the paralysis of the world economy^5^ by brutally unveiling the gap between global economic interdependence and nation-state governance. No other response to a disaster led to the home confinement of more than half of the world’s population. No other risk management response raised so many novel legal,^6^ ethical,^7^ moral^8^ and political issues. No other pandemic has been covered continually and in real time by a 24-hour news cycle, often reporting conflicting information simultaneously, and further amplified by social media users.^9^ No other crisis has suddenly reshaped our individual and collective idea of risk. Ultimately, no other occurrence carries the potential to disrupt our economic, legal, democratic, social and cultural systems as well as the relationships among them to the same extent.

All of the above makes COVID-19 qualitatively different from any of the six outbreaks previously identified by the World Health Organization as pandemics (“Public Health Emergency of International Concern”, hereinafter PHEIC): influenza, polio, Ebola,^10^ Zika, Middle East respiratory syndrome (MERS)^11^ and severe acute respiratory syndrome (SARS)^12^.

COVID-19 represents not only one of the greatest natural and economic disasters in human history, but also a rich case study of governments’ emergency responses. As such, it is a test bed for virtually all disciplines – be they natural sciences, social sciences or humanities.^13^ This is all the more true for risk research and regulatory theories in a world increasingly shaped by transboundary, uncertain manufactured and natural risks. Ultimately, as Ulrich Beck observed, “risks cannot be understood outside their materialization in particular mediation, be they scientific, political, economic or popular”.^14^

How has a mature, self-aware “world risk society”^15^ been caught by surprise by an essentially foreseeable event like a novel coronavirus (nCoV)?^16^ If the virus spreads transnationally, why then have nation-states favoured national solutions instead of exploring common multilateral solutions? Why did countries – that were initially unwilling to cooperate and adopted different, sometimes competing, approaches to the outbreak^17^ – eventually converge on a common yet uncoordinated risk suppression policy translating into generalised, indiscriminate “social distancing”? What are the actual social, economic and distributive costs entailed by such an uncoordinated, but essentially similar approach? What other major, sometimes excruciating “risk versus risk trade-offs”^18^ are involved in such a common approach (e.g. home confinement versus higher domestic violence; school closure versus higher risk of exposure of grandparents)? Why has the public not been informed about the excruciating trade-offs faced by decision-makers, nor about the criteria used to reach their conclusions?^19^ What liability might countries have incurred due to negligence or omission in their COVID-19 response?^20^ Why did data not play a greater role in predicting and countering the spread of the disease?^21^ Why were existing digital tools not quickly turned into epidemic-monitoring instruments – and without intruding into people’s lives?^22^ How are we to explain the high degree of public acceptance of unprecedented freedom-restrictive coercive measures, such as lockdowns, which have been essentially imposed on – and remained unexplained to – the public? Why have existing global,^23^ regional and national emergency preparedness and response mechanisms proved – once more – so useless, letting governments remain in the driving seat?^24^ Why have the European Union – one of the major epicentres of the pandemic – and its members proven incapable of coordinating their respective actions while curbing the spread of the virus?^25^ What if – as the spread of the virus continues to unfold and some knowledge gaps are filled – the social and economic cost of distancing will outweigh the health benefits sought? What if “state emergency governance” crystallises within governments and becomes the preferred way of doing business in the post-COVID-19 world?^26^

While it is obviously too early to come to definitive conclusions, the contributions collected – in record time – in this special issue of the *European Journal of Risk Regulation* attempt to provide some initial responses to some of these hard questions. They do so by unpacking the complex combination of a disease pandemic combined with flawed, or at least unprepared, national, regional and international institutions operating in a geopolitically shattered world.

There are several provisional explanations for the global suboptimal response to an essentially foreseeable outbreak^27^ such as this pandemic. They include a Knightian^28^ unmeasurable uncertainty^29^ surrounding SARS-CoV-2^30^ and the disease it causes;^31^ limited systematic evaluation of the effectiveness of non-pharmaceutical interventions;^32^ inadequate diagnostic testing capacity limiting our understanding of the disease’s reproduction, which in turn affects the choice of the risk responses;^33^ the ensuing difficulty in identifying and gauging major trade-offs behind any risk intervention; the lack of an effective global alarm response capacity in an highly interdependent yet geopolitically volatile world;^34^ widespread unpreparedness of our respective health systems, in particular their inability to cater for a surge in capacity^35^; the inability to mobilise the unprecedented wealth of data collected today to counter the virus due to the absence of a data governance and data-sharing culture as well as public–private infrastructure;^36^ and the lack of evidence-based communication^37^ to – and engagement with – the public at a time of unprecedented disinformation.^38^

The COVID-19 scenario contains all of the ingredients of a textbook case of risk regulation, in which regulators attempt to stabilise decision-making by mobilising a variety of bodies of knowledge guiding their action.^39^ The ingredients include the effort of turning uncertainties into probabilities through scientific assessment methodologies;^40^ the difficult interplay between science and politics, as well as between democracy and evidence-based public policy, when responding to risk through risk management;^41^ the deficient and typically conflicting guidance of precautionary thinking and cost–benefit analysis;^42^ the need to factor in people’s perceptions of risk and responses to it,^43^ especially when communicating about it;^44^ and, ultimately, the inescapable coexistence of societal risks (ie the health threat posed by the virus) with institutional risks (ie the risk of policy failure in relation to a societal risk).

In other words, despite being a qualitatively different pandemic from those witnessed over the last century, there is little new in COVID-19, at least from a risk regulation perspective. These questions and scenarios are part of any government’s attempt to routinise decisions on how to respond to risk through the help of agencies, expert committees and dedicated emergency rapid response mechanisms. The challenges raised by COVID-19 can therefore be seen as the “bread and butter” of our discipline.

Against this backdrop, one may therefore reasonably expect that risk regulation may come up with responses that not only save lives, but also livelihoods, and do also in emergency situations.

Yet, when measured against this benchmark, the global, dominant response to COVID-19 may appear suboptimal. While the generalised, indiscriminate social distancing policy may have had net benefits when it was initially implemented^45^ – contributing to slowing down the spread of the virus while saving tens of thousands of lives^46^ – it has been showing its limitations over time, as the restrictive measures led to major social and distributive costs.^47^ Moreover, as states of emergency are declared throughout the world in response to the spread of COVID-19, concerns arise as to the use – and potential abuse – of power in a time of crisis.^48^ As COVID-19 was spreading, many prominent nationalist populists – from Duterte to Bolsonaro, Trump to Johnson – started off by dismissing fears. They appealed to gut feeling and “common sense” over science and fact, but ultimately – as the COVID-19 outbreak accelerated – they were forced to acknowledge the seriousness of the situation and act.^49^ Yet their attitude inherently weakened the case for those measures, as they were initially dismissed as overreaction. In particular, it slowed down their social acceptance, and even led to the adoption of unscientific interventions such as travel bans.^50^

The resulting global response to COVID-19, based on nation-state-based, uncoordinated and often times politicised solutions, already calls for some self-reflection.

To what extent has risk regulation proved inadequate, incapable and even unfit to govern the challenges raised by COVID-19? Or was this the outcome of decision-makers’ fault insofar as their response failed to follow established conventional risk analysis frameworks and failed to embrace the full potential of data? Or, ultimately, can the magnitude of the emergency surrounding this outbreak justify by itself such a suboptimal outcome?^51^

Be that as it may, this pandemic has been testing the ability of risk regulation, both as a discipline and as a practice of government. Risk regulation is not only expected to provide immediate, cost-effective responses, but also to be able to co-create these responses – through participatory mechanisms – when addressing a legitimate public health threat with an unknown trajectory, and to be able to do so in a situation of emergency.

## Conclusions

As this Special Issue clearly suggests, we have been thinking about how to govern risks, including disasters – such pandemic diseases^52^ – for a long time. Over the last decade, this journal has been providing its own contribution by publishing a variety of timely symposia – such as those dedicated to the volcanic ash eruption in Iceland^53^, the BP Deepwater Horizon oil spill,^54^ the L’Aquila earthquake^55^ and now the global risk regulatory response to COVID-19 – and featuring original research articles striving to advance the state of research.^56^

Risk regulation is uniquely well situated to play a prominent role in advancing government’s ability to tame disasters. By mobilising many bodies of knowledge and blending their insights, it can better inform our understanding of risk scenarios and – once those unveil – guide decision-makers when facing *inter alia* excruciating trade-offs and communicating them to the public.

However, if we have learned from previous experiences that even foreseeable challenges can be inherently hard to prepare for,^57^ COVID-19 is no exception.

Once more, the handling of this pandemic brutally unveiled the gap between the sophistication of risk regulation – its epistemic fluency,^58^ established principles and analytical frameworks – and the improvisation of an inherently undisciplined political process tasked to govern, even in emergencies.

To close this gap, we need to rethink not only how to make risk regulation principles relevant and actionable, but also how to make them stick with decision-makers and bureaucracies both within and across states. Unless decision-makers are made accountable to the public for their reliance of, or departure from, well-established risk regulatory principles and frameworks, risk decision-making will be left to extemporisation and inevitably produce unscientific outcomes.^59^

Failing to do so will let COVID-19 go down in history as yet another major disaster occurrence with no learnings attached. It will not be another tweak to the design of emergency preparedness plans, be they global or regional, or the setting up of another emergency body or mechanism that will fully capitalise on what COVID-19 revealed to us.

As new transboundary disasters – from bioterrorism to climate change – loom on the horizon, neither the world nor our discipline can afford to let another crisis go wasted.

*Au travail!*

## Footnotes

1 Coronavirus disease (COVID-19) is caused by SARS-CoV-2 and represents the causative agent of a potentially fatal disease that is of great global public health concern. See, eg, HA Rothan and SN Byrareddy, “The Epidemiology and Pathogenesis of Coronavirus Disease (COVID-19) Outbreak” (2020) 109 Journal of Autoimmunity 102433. New coronaviruses appear to emerge periodically in humans, mainly due to the high prevalence and wide distribution of coronaviruses, the large genetic diversity and frequent recombination of their genomes and the increasing of human–animal interface activities.

2 S Morse, “Pandemic Influenza: Studying the Lessons of History” (2007) 104 Proceedings of the National Academy of Sciences of the United States of America 7313.

3 S Morse, RL Garwin and PJ Olsiewski, “Next Flu Pandemic: What to Do Until the Vaccine Arrives?” (2006) 314 Science 929.

4 See, eg, JM Barry, *The Great Influenza:* *The Story of the Deadliest Pandemic in History* (London, Penguin 2006); AW Crosby, *America’s Forgotten Pandemic: The Influenza of 1918* (Cambridge, Cambridge University Press 1989).

5 See, eg, IMF, “World Economic Outlook, April 2020: The Great Lockdown”. See also, eg, AKL Milne, “A Critical COVID-19 Economic Policy Tool: Retrospective Insurance”, 21 March 2020; A Tooze, “The Shock of Coronavirus Could Split Europe – Unless Nations Share the Burden”, *The Guardian*, 25 March 2020.

6 For an initial assessment of the most pressing legal issues the pandemic has raised, from a US perspective, see, eg, K Pistor, “Law in the Time of COVID-19” (2020) *<* https://scholarship.law.columbia.edu/books/240>.

7 See, eg, WHO Working Group on Ethics & COVID-19 tasked to develop advice on key ethical questions that Member States need to address.

8 J Auters, “How Coronavirus Is Shaking Up the Moral Universe”, *Bloomberg*, 29 March 2020.

9 S Ratzan, L Gostin, N Meshkati, K Rabin and R Parker, “COVID-19: An Urgent Call for Coordinated, Trusted Sources to Tell Everyone What They Need to Know and Do”. *NAM Perspectives*. Commentary, National Academy of Medicine, Washington, DC (2020) <https://doi.org/10.31478/202003a>.

10 In West Africa and in the Democratic Republic of Congo.

11 MERS, which appeared on the Saudi peninsula after the International Health Regulations revision, was never officially designated a PHEIC, despite its international spread.

12 SARS was not designated a PHEIC as it occurred before the revision of the World Health Organization (WHO) International Health Regulations.

13 J Kagan, *The Three Cultures: Natural Sciences, Social Sciences and the Humanities in the 21st Century* (Cambridge, Cambridge University Press 2009).

14 U Beck, *World Risk Society* (Cambridge, Polity Press 1999).

15 Sociologists have been telling us for some time that we live in a “risk society”, in which society is increasingly occupied by the management of risks it has itself created. See A Giddens, *The Consequence of Modernity* (Cambridge, Cambridge University Press 1990); U Beck, *Risk Society: Towards a New Modernity* (Thousand Oaks, CA, Sage 1992); A Giddens, “Risk and Responsibility” (1999) 62 Modern Law Review 1.

16 A variety of sources have predicted over the years a pandemic presenting the features of COVID-19. Most virologists expected as “inevitable at least one pandemic in this century”; see Morse, supra, note 2. Most recently, a 2019 report by the Global Preparedness Monitoring Board, published under the auspices of the WHO, made a compelling case for preparedness: “The world is at acute risk for devastating regional or global disease epidemics or pandemics that not only cause loss of life but upend economies and create social chaos”; see “A World at Risk: Annual Report on Global Preparedness for Health Emergencies”, September 2019. The WHO published its first Disease Outbreak News on the new virus on 5 January 2020. It contained a risk assessment and advice, and reported on what China had told the WHO about the status of patients and the public health response on the cluster of pneumonia cases in Wuhan: <https://www.who.int/csr/don/05-january-2020-pneumonia-of-unkown-cause-china/en>. The European Centre for Disease Prevention and Control (ECDC) reporting on 25 January 2020: “In light of the currently available information, ECDC considers that the potential impact of 2019-nCoV outbreaks is high and further global spread is likely”. The US Secretary of Health and Human Services declared the SARS-CoV-2 virus a US public health emergency on 31 January *2020*, but in September 2019, the US Council of Economic Advisers cautioned the Trump Administration about the likelihood of a flu pandemic on the scale of the influenza epidemic 100 years ago: <https://www.whitehouse.gov/wp-content/uploads/2019/09/Mitigating-the-Impact-of-Pandemic-Influenza-through-Vaccine-Innovation.pdf>.

17 For an insightful account in this Special Issue, see A Sibony, “The UK COVID-19 Response: A Behavioural Irony?” (2020) European Journal of Risk Regulation doi: 10.1017/err.2020.22. On the Swedish approach and its major sanitary and legal implications, see E Herlin-Karnell, *“*Corona and the Absence of a Real Constitutional Debate in Sweden”, *VerfBlog,* 10 April 2020 <https://verfassungsblog.de/corona-and-the-absence-of-a-real-constitutional-debate-in-sweden> and “Why Swedes Are Not Yet Locked Down?” *The Economist*, 4 April 2020.

18 The expression “risk versus risk trade-offs” is used to designate cases where measures implemented to mitigate one risk to human health or the environment knowingly or unintentionally created another new risk equally or more problematic than the original risk. See, eg, J Graham and J Wiener, *Risk vs. Risk: Tradeoffs in Protecting Health and the Environment* (Cambridge, MA, Harvard University Press 1995).

19 See, in this Special Issue, S Chakraborty, “How Risk Perceptions, Not Evidence, Have Driven Harmful Policies on COVID-19” (2020) European Journal of Risk Regulation doi: 10.1017/err.2020.37.

20 See, in this Special Issue, L Bergkamp, “State Liability for Failure to Control the COVID-19 Epidemic: International and Dutch Law” (2020) European Journal of Risk Regulation doi: 10.1017/err.2020.21.

21 On the incipient institutionalisation of data use in public health policies and its perils, see, in this Special Issue, A Spina and C Cattuto, “The Institutionalisation of Digital Public Health: Lessons Learned from the COVID-19 App” (2020) European Journal of Risk Regulation doi: 10.1017/err.2020.47.

22 For an overview on how mobile phone data can guide government and public health authorities in determining the best course of action to control the COVID-19 pandemic and in assessing the effectiveness of control measures such as physical distancing, see N Oliver et al, “Mobile Phone Data and COVID-19: Missing an Opportunity?” Working paper available at <https://arxiv.org/pdf/2003.12347.pdf>.

23 Fifteen years ago, the WHO undertook a major revision of the International Health Regulations – the global framework for responding to outbreaks designated as a PHEIC – trying to fix perceived shortcomings in the global response experienced during the SARS outbreak in 2003. See WHO, “Fifty-Eighth World Health Assembly. Revision of the International Health Regulation”, WHA 58.3; agenda item 3.1:23, May 2005. The revision expanded its scope of application, the obligations of States Parties and WHO’s powers to respond to public health emergencies. See, eg, K Wilson, JS Brownstein and DP Fidler, “Strengthening the International Health Regulations: Lessons from the H1N1 Pandemic” (2010) 25 Health Policy and Planning 505. See also, in this Special Issue, M Broberg, “A Critical Appraisal of the World Health Organization’s International Health Regulations (2005) in Times of Pandemic: It Is Time for Revision” (2020) European Journal of Risk Regulation doi: 10.1017/err.2020.26.

24 Regarding the question on whether national decision-making or a supranational approach is more effective or appropriate in the context of COVID-19, see, in this Special Issue: M Dobbs, “National Governance of Public Health Responses in a Pandemic?” (2020) European Journal of Risk Regulation doi: 10.1017/err.2020.39.

25 For a reconstruction and initial evaluation of the European response to COVID-19, see, in this Special Issue, A Renda and R Castro, “Towards Stronger EU Governance of Health Threats after the COVID-19 Pandemic” (2020) European Journal of Risk Regulation doi: 10.1017/err.2020.34; KP Purnhagen, A de Ruijter, ML Flear, TK Hervey and A Herwig, “More Competences than You Knew? The Web of Health Competence for Union Action in Response to the COVID-19 Outbreak” (2020) European Journal of Risk Regulation doi: 10.1017/err2020.35; AM Pacces and M Weimer, “From Diversity to Coordination: A European Approach to COVID-19” (2020) European Journal of Risk Regulation doi: 10.1017/err.2020.36; A Alemanno, “The European Response to COVID-19: From Regulatory Emulation to Regulatory Coordination?” (2020) European Journal of Risk Regulation doi: 10.1017/err.2020.44. For a specific aspect of the EU’s response related to personal protective equipment see, in this Special Issue, LA Jiménez and M Eliantonio, “Mask, Gloves, Exports Licences and Composite Procedures: Implementing Regulation 2020/402 and the Limelight of Accountability” (2020) European Journal of Risk Regulation doi: 10.1017/err.2020.28.

26 See, in this Special Issue, A Spadaro, “COVID-19: Testing the Limits of Human Rights” (2020) European Journal of Risk Regulation doi: 10.1017/err.2020.27; see also, more broadly, RL Tsai, “Manufactured Emergencies” (2020) 129 The Yale Law Journal 590.

27 J Platjes, J Harvey and L Rayman-Bacchus, “COVID-19 – Reflections on the Surprise of Both an Expected and Unexpected Event” (2020) 4 The Central European Review of Economics and Management 149.

28 In Frank Knight’s seminal work, uncertainty must be distinguished from risk insofar as – unlike the latter – it is not susceptible to quantitative formula. See F Knight, *Risk, Uncertainty, and Profit* (Boston, MA, Hart, Shaffner and Marx 1921).

29 For an overview of the “knowledge gaps” of COVID-19, see MM Hossain, “Current Status of Global Research on Novel Coronavirus Disease (COVID-19): A Bibliometric Analysis and Knowledge Mapping”, 2 April 2020 <http://dx.doi.org/10.2139/ssrn.3547824>.

30 COVID-19 is the most serious respiratory virus since the 1918 H1N1 influenza pandemic. See NM Ferguson et al, “Impact of Non-Pharmaceutical Interventions (NPIs) to Reduce COVID-19 Mortality and Healthcare Demand”, Imperial College London, 16 March 2020 <https://doi.org/10.25561/77482>.

31 A Wilder-Smith, CJ Chiew and VJ Lee, “Can We Contain the COVID-19 Outbreak with the Same Measures as for SARS?” (2020) The Lancet Infectious Diseases <https://www.thelancet.com/journals/laninf/article/PIIS1473-3099(20)30129-8/fulltext>.

32 For an attempt at a systematic review of the evidence base for the effectiveness of multiple mitigation measures, see MW Fong, “Nonpharmaceutical Measures for Pandemic Influenza in Nonhealthcare Settings” (2020) 26 Emerging Infectious Diseases 961.

33 See, in this Special Issue, M Morvillo, “I Just Can’t Get Enough (of Experts): The Numbers of COVID-19 and the Need for a European Approach to Testing” (2020) European Journal of Risk Regulation doi: 10.1017/err.2020.41.

34 Broberg, supra, note 23. See also, eg, GL Burci, “The Outbreak of COVID-19 Coronavirus: Are the International Health Regulations Fit for Purpose?” *EJIL Talk*, 27 February 2020.

35 For a short analysis of COVID-19’s impact on healthcare resources in the UK, see, in this Special Issue, S Germain, “Will COVID-19 Mark the End of an Egalitarian National Health Service?” (2020) European Journal of Risk Regulation doi: 10.1017/err.2020.33.

36 Spina and Cattuto, supra, note 21.

37 Chakraborty, supra, note 19.

38 JY Cuan-Baltazar, MJ Muñoz-Perez, C Robledo-Vega, MF Pérez-Zepeda and E Soto-Vega, “Misinformation of COVID-19 on the Internet: Infodemiology Study” (2020) 6(2) JMIR Public Health and Surveillance e18444.

39 J Black, “The Role of Risk in *Regulatory* Processes”, in *The Oxford Handbook of Regulation* (Oxford, Oxford University Press 2010) pp 302–48.

40 M Douglas, *Risk Acceptability According to the Social Sciences* (London, Routledge 1985).

41 S Jasanoff, *The Fifth Branch: Science Advisers as Policymakers* (Cambridge, MA, Harvard University Press 1990).

42 See, eg, JB Wiener, “Precaution in a Multi-Risk World” in *Human and Ecological Risk Assessment: Theory and Practice* (Hoboken, NJ, Wiley-Blackwell 2002) pp 1509–31; CR Sunstein, “Cost–Benefit Analysis and the Environment” (2005) 115(2) Ethics 351.

43 See, eg, P Slovic, B Fischhoff and S Lichtenstein, “Regulation of Risk: A Psychological Perspective” in R Noll (ed.), *Regulatory Policy and the Social Sciences* (Berkeley, University of California Press 1985) pp 241–78; P Slovic, “Perception of risk” (1987) 236(4799) Science 280.

44 See, eg, MG Morgan, B Fischhoff, A Bostrom and CJ Atman, *Risk Communication: A Mental Models Approach* (Cambridge, Cambridge University Press 2002); DC Glik, “Risk Communication for Public Health Emergencies” (2007) 28(1) Annual Review of Public Health 33.

45 For a US study showing that the benefits from strict social distancing in terms of lives saved significantly outweigh the economic costs, see M Greenstone and V Nigam, “Does Social Distancing Matter?” University of Chicago, Becker Friedman Institute for Economics Working Paper No. 2020-26, 30 March 2020 <https://ssrn.com/abstract=3561244>. See also C Sunstein, “Coronavirus: Lockdowns Look Smart under Cost–Benefit”, *Bloomberg*, 26 March 2020. In Europe, no cost–benefit analysis was publicly made available at the time of writing, neither at the national nor EU level. For epidemiological modelling, see NM Ferguson et al, supra, note 30.

46 See, eg, H Tian et al, “The Impact of Transmission Control Measures during the First 50 Days of the COVID-19 Epidemic in China” (2020) medRxiv preprint <https://doi.org/10.1101/2020.01.30.20019844>; M Chinazzi et al, “The Effect of Travel Restrictions on the Spread of the 2019 Novel Coronavirus (COVID-19) Outbreak” (2020) Science <https://science.sciencemag.org/content/368/6489/395.long>. See also V Alfano and S Ercolano, “Shut It Down: A Cross Country Panel Analysis on the Efficacy of Lockdown Measures” (2020) medRxiv preprint <https://doi.org/10.1101/2020.04.12.20062695>.

47 See. eg, A Glover, J Heathcote, D Krueger and J Rios-Rull, “Health versus Wealth: On the Distributional Effects of Controlling a Pandemic”, London, Centre for Economic Policy Research (2020) <https://cepr.org/active/publications/discussion_papers/dp.php?dpno=14606>; M Andersen, “Early Evidence on Social Distancing in Response to COVID-19 in the United States”, 6 April 2020, <http://dx.doi.org/10.2139/ssrn.3569368≥; for an anthropological critique of social distancing, see NJ Long, “From Social Distancing to Social Containment: Reimagining Sociality for the Coronavirus Pandemic” (2020) Medicine Anthropology Theory; for an initial assessment of the democratic costs of COVID-19, see F Bieber, “Authoritarianism in the Time of the Coronavirus”, *Foreign Policy*, 30 March 20202; for an initial assessment of the impact on inequalities, see F Ahmed, N Ahmed, C Pissarides and J Stiglitz*, “*Why Inequality Could Spread COVID-19” (2020) The Lancet <https://www.thelancet.com/pdfs/journals/lanpub/PIIS2468-2667(20)30085-2.pdf>.

48 J Grogan,” COVID 19 and States of Emergency: Introduction & List of Country Reports”, *Verfassungsblog*, April 2020 <https://verfassungsblog.de/introduction-list-of-country-reports>.

49 For a thoughtful account on how populist leaders inherently reject the analytical framework offered by risk regulation and its technocratic solutions, see J Cliff, “How Populist Leaders Exploit Pandemics”, *New Statesman*, 18 March 2020.

50 Chinazzi et al, supra, note 46.

51 For an initial attempt at theorising the concept of emergency risk regulation and identifying its peculiar features, see A Alemanno (ed.), *Governing Disasters: The Challenges of Emergency Risk Regulation* (Cheltenham, Edward Elgar 2011), building upon some pre-existing contributions coming from a variety of disciplines, such as political science (see, eg, C Ansell, A Bojn and A Keller, “Managing Transboundary Crises: Identifying the Building Blocks of an Effective Response System” (2010) 18(4) Journal of Contingencies and Crisis Management 195), law (see, eg, RA Posner, *Catastrophe: Risk and Response* (New York, Oxford University Press 2004); CR Sunstein, *Worst-Case Scenarios* (Cambridge, MA, Harvard University Press 2007; DA Farber and M Fauré, *Disaster Law* (Cheltenham: Edward Elgar 2010)), management (see, eg, LT Drennan and A McConnell, *Risk and Crisis Management in the Public Sector* (New York: Routledge 2007); P Lagadec, *States of Emergency: Technological Failures and Social Destabilization* (London: Butterworth-Heinemann 1990)); and sociology (see, eg, B Hutter, *Anticipating Risks and Organising Risk Regulation* (Cambridge, Cambridge University Press 2010)).

52 For an authoritative collection focusing essentially on natural disaster as opposed to “slow-moving” disasters, such as disease epidemics, terrorism and climate change, see, eg, Farber and Fauré, supra, note 51.

53 “The European Regulatory Response to the Volcanic Ash Crisis between Fragmentation and Integration” (2010) 1(2) European Journal of Risk Regulation.

54 R Tardiff et al, “Mini-Symposium on the Deepwater Horizon Oil Spill” (2010) 1(3) European Journal of Risk Regulation.

55 “Symposium on the L’Aquila Seven: Re-Establishing Justice after a Natural Disaster” (2014) 4(2) European Journal of Risk Regulation.

56 See, eg, M-V Florin, “Dealing with the Challenge of Evidence-based Decision-making in Situations of Uncertainty and Emergency” (2014) 5(3) European Journal of Risk Regulation 303; T Binder, A Karagianni and M Scholten, “Emergency! But What about Legal Protection in the EU?” (2018) 9(1) European Journal of Risk Regulation 99; M Rizzi, “Vaccines for Pandemic and Epidemic Diseases: Towards Defining the Space of EU Public Health between Security Policy and a Transnational Market” (2017) 8(4) European Journal of Risk Regulation 686.

57 See, eg, DA Rose, S Murthy, J Brooks and J Bryant, “Evolution of Public Health Emergency Management as a Field of Practice” (2017) 107(S2) American Journal of Public Health S126.

58 By epistemic fluency, I mean the capacity to recognise different kinds of knowledge and to work flexibly with different ways of knowing, which is inherent to risk regulation, both as a discipline and a practice.

59 One of the most visible illustration at the time of COVID-19 is travel bans; see, eg, Chinazzi et al, supra, note 46.

